# Gene flow and natural selection shape spatial patterns of genes in tree populations: implications for evolutionary processes and applications

**DOI:** 10.1111/eva.12316

**Published:** 2015-11-03

**Authors:** Victoria L. Sork

**Affiliations:** ^1^ Department of Ecology and Evolutionary BiologyUniversity of CaliforniaLos AngelesCAUSA; ^2^ Institute of Environment and SustainabilityUniversity of CaliforniaLos AngelesCAUSA

**Keywords:** gene flow, landscape genomics, local adaptation, phylogeography, pollen dispersal, population differentiation, *Quercus*, seed dispersal

## Abstract

A central question in evolutionary biology is how gene flow and natural selection shape geographic patterns of genotypic and phenotypic variation. My overall research program has pursued this question in tree populations through complementary lines of inquiry. First, through studies of contemporary pollen and seed movement, I have studied how limited gene movement creates fine‐scale genetic structure, while long‐distance gene flow promotes connectivity. My collaborators and I have provided new tools to study these processes at a landscape scale as well as statistical tests to determine whether changes in landscape conditions or dispersal vectors affect gene movement. Second, my research on spatial patterns of genetic variation has investigated the interacting impacts of geography and climate on gene flow and selection. Third, using next‐generation genomic tools, I am now studying genetic variation on the landscape to find initial evidence of climate‐associated local adaptation and epigenetic variation to explore its role in plant response to the climate. By integrating these separate lines of inquiry, this research provides specific insight into real‐world mechanisms shaping evolution in tree populations and potential impacts of landscape transformation and climate change on these populations, with the prospective goal of contributing to their management and conservation.

## Introduction

“In considering the distribution of organic beings over the face of the globe, the first great fact which strikes us is, that neither the similarity nor the dissimilarity of the inhabitants of various regions can be accounted for by their climatal and other physical conditions… The dissimilarity of inhabitants of different regions may be attributed to modification through natural selection… The degree of dissimilarity will depend on the migration of the more dominant forms of life from one region into another… Thus, the high importance of barriers comes into play by checking migration; as does time for the slow process of modification through natural selection”. (Darwin 1859)

Understanding the relative contributions of gene flow and natural selection to geographical patterns of natural variation has been a recurring theme of evolutionary biology. Darwin goes back and forth between the two processes in *The Origin of Species*, and those who have followed (Fisher, Wright, Mayr, Stebbins—to name a few) have continued to grapple with the roles of these countervailing forces. Gene flow and natural selection are two central, and usually opposing, evolutionary forces: gene flow distributes, homogenizes, and maintains genetic variation that can act as the ‘stuff of evolution’, while natural selection reduces genetic variation to the variants that favor survival and reproduction. For tree species, which are often highly outcrossed with extensive gene flow, genetic drift plays a lesser role in their population genetics, except in small or substantially isolated populations (Ledig 1988, 1992; Ellstrand and Elam 1993). Effective dispersal mechanisms maintain connectivity of populations, except where they encounter a barrier. Nonetheless, within the species range, gene flow has a decay rate that will create genetic isolation by distance, setting up a pattern of genetic variation (genetic structure) within and among populations (Wright 1943; Slatkin 1985). Once a gene moves and a plant becomes established, it must survive where it lands, and long‐lived plants will experience selection throughout life, with local ecological forces creating spatial genetic structure across the landscape (Bradshaw 1972). Endler (1973) proposed that selection is often so strong that the ‘swamping out’ effects of gene flow should be negligible. Of course, the relative impact of selection versus gene flow will vary across loci, depending on whether or not they experience selection.

One way to understand how the dynamics of gene movement create genetic structure is to analyze contemporary gene movement through pollen and seeds (Levin 1981; Hamrick 1987; Sork et al. 1999) and assess their impacts on local neighborhood size, which is the effective number of interbreeding individuals (Wright 1946; Crawford 1984). Numerous studies have used paternity analysis to assess the distance of pollen‐mediated gene dispersal and the shape of the dispersal kernel (e.g., Adams et al. 1992; Smouse et al. 1999; Streiff et al. 1999; Burczyk et al. 2002; Slavov et al. 2005; Oddou‐Muratorio et al. 2006; Meagher 2007; Pluess et al. 2009). Alternatively, one can use the genetic composition of the pollen pool, such as TwoGener and related methods (e.g., Smouse et al. 2001; Austerlitz et al. 2004; Smouse and Sork 2004; Smouse and Robledo‐Arnuncio 2005; Robledo‐Arnuncio et al. 2006), to document contemporary pollen‐mediated gene dispersal, even when paternity analysis is not possible. Studies using both approaches often illustrate how well pollen flow promotes gene flow. In contrast, studies documenting seed‐mediated gene dispersal using maternity analysis and alternative methods generally demonstrate more restricted gene movement (e.g., Hamrick et al. 1993; Godoy and Jordano 2001; Garcia et al. 2005; Grivet et al. 2005), with notable exceptions (e.g., Nason and Hamrick 1997; Aldrich and Hamrick 1998; Dick et al. 2003; Karubian et al. 2010). Various types of pollen and seed dispersal vectors have differential impacts on fine‐scale genetic structure that can be assessed through spatial autocorrelation analysis (Sokal and Oden 1979; Loiselle et al. 1995; Epperson 2003; Vekemans and Hardy 2004). Resulting patterns are consistent with observations of contemporary gene movement, such that high movement reduces fine‐scale structure and vice versa (Hamrick and Loveless 1986; Hardy et al. 2006; Dick et al. 2008). A few studies have been able to examine both processes simultaneously and show that the contribution of seed dispersal in particular plays a critical role in the genetic structure of the seedlings populations (e.g., Garcia et al. 2007; Grivet et al. 2009; Ottewell et al. 2012; Sork et al. 2015), thus shaping the opportunity for the natural selection to create local adaptation.

Until recently, studying adaptive genetic variation in natural populations has not been as feasible as studying gene flow. However, next‐generation sequencing now makes it possible to identify the single‐nucleotide polymorphisms (SNPs) that flag candidate genes underlying traits experiencing selection created by local environments, even for nonmodel species without reference genomes (Davey et al. 2011; Sork et al. 2013). For locally adapted genetic variation, allele frequencies at these SNPs might exhibit significantly higher population differentiation than other sampled SNPs, after controlling for demographic history (Davey et al. 2011). All the same, the proper designs and statistical tests to avoid false positives are still a work in progress (Lotterhos and Whitlock 2014, 2015). Another way to obtain evidence for local adaptation would be to examine the association between allele frequencies with environmental variables (Luikart et al. 2003; Nielsen 2005; Coop et al. 2010; Strasburg et al. 2012). These findings can be combined with other genomic and quantitative genetic approaches to determine the gene functions or phenotypes that might be under selection (Stapley et al. 2010; Neale and Kremer 2011; Andrew et al. 2013; Sork et al. 2013). For evolutionary ecologists and conservation biologists, it is exciting that we now have genomic tools to study the adaptive genetic variation in natural populations.

Understanding the interaction of gene flow and selection in natural populations is fundamental to many applied evolutionary questions. The field of landscape genetics has emerged in response to a need to understand the impact of fragmentation and other landscape changes on gene flow and the preservation of genetic variation (Sork et al. 1999; Manel et al. 2003; Sork and Waits 2010). Much of population genetic theory assumes null environments and equilibrium conditions, but the rapidly changing landscapes created by human perturbations make those assumptions invalid. Moreover, conservation scientists focus on the impact of those changes (Ledig 1992; Ellstrand and Elam 1993; Young et al. 1996; Nason and Hamrick 1997; Aguilar et al. 2008), thus, requiring new methods of estimating their genetic consequences at a landscape scale. In addition, to establish the credibility of conservation science, we have needed improved empirical tests to determine whether human disturbance has significantly affected populations. The concern of environmental scientists about the impact of anthropogenic landscape change on genetic diversity and gene flow has now been extended to apprehension about the effect of climate change on the sustainability of populations (Parmesan 2006; Aitken et al. 2008; Sork et al. 2010; Anderson et al. 2012; Kremer et al. 2014). Through models of future climate (e.g., Cayan et al. 2008), it will be possible to identify the geographic regions with a high probability of climate change in order to specify the areas of conservation priority and then examine populations within these regions to assess their vulnerability.

**Figure 1:**
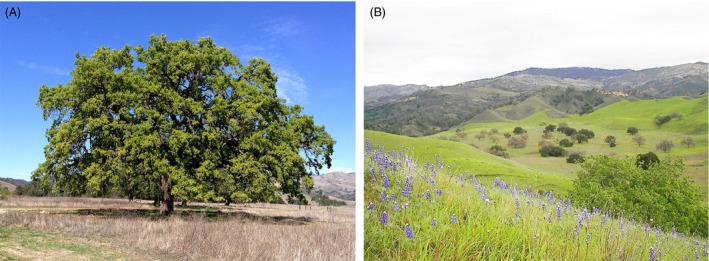
(A) Photo of adult *Quercus lobata* Née. (Photo by Andy Lentz *©*) (B) Landscape view of typical oak savanna habitat with scattered large trees of *Q. lobata* (leafless canopies) and *Q. agrifolia* (green canopies) taken at UC Santa Barbara Sedgwick Reserve, Santa Ynez Valley, Santa Barbara Co., California, USA. (Photo by Delphine Grivet ©).

Over the last twenty plus years, my research has focused on gene flow and geographical patterns of genetic variation at different temporal and spatial scales. Several tree species in different ecosystems have been studied with the goals of understanding the fundamental evolutionary processes of gene flow and selection and applying that knowledge to the development of conservation and resource management practices and strategies. To generate a general framework for trees, I selected the iconic California endemic oak, *Quercus lobata* Née, as our focal study system (Fig. 1). *Q. lobata* is a foundation species for biodiversity in the ecosystems it defines, and it has experienced significant reduction in its geographic coverage since the arrival of Europeans (Kelly et al. 2005; Whipple et al. 2011). Pavlik et al. (1995) described valley oak as the ‘monarch of California oaks by virtue of its size, age, and beauty’. This research has generated empirical data and new methods to address key concepts through three lines of inquiry:

1. *Contemporary gene flow at a landscape scale*. Studying ongoing pollen‐ and seed‐mediated gene movement provides valuable insight into the intrinsic properties of the dispersal vectors and the influences of human disturbance on the distribution of genetic variation within and among populations.
2. *Geographic patterns of genetic variation*. Spatial patterns of genetic variation across the species range, especially when combined with environmental data, can clarify the contributions of gene flow, historical demographic events, and the environment on the distribution of genetic variation.
3. *Landscape genomics of adaptive genetic variation*. Landscape genomics provides the basis for studying adaptive genetic variation by geolocating sequence data, flagging outliers associated with climate, and identifying candidate genes. This approach provides initial evidence of local adaptation in climate‐associated genes that can be complemented with studies of phenotypes in common gardens and experimental studies of gene expression in response to environmental treatments.

## Background and initial drivers

**Figure 2:**
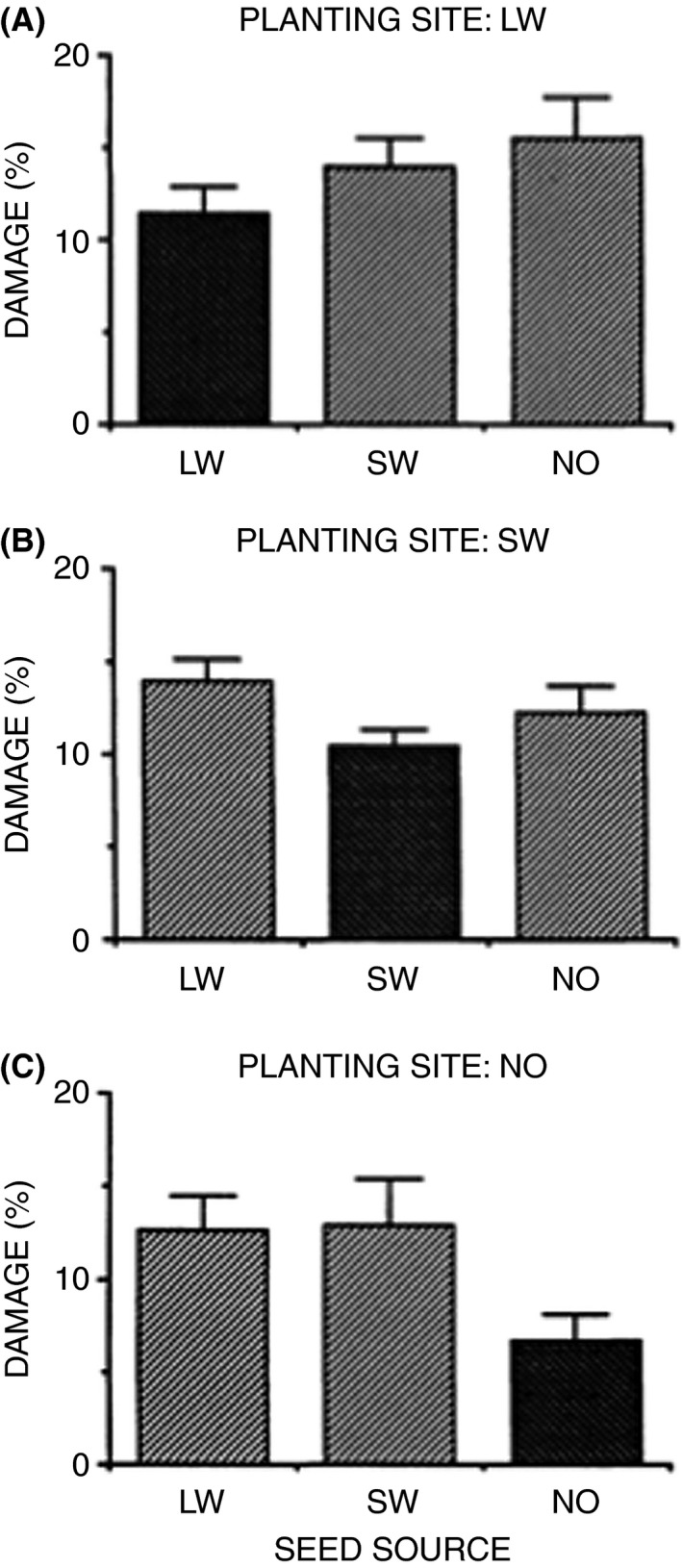
Percent leaf damage by herbivores on *Quercus rubra* seedlings grown from seed derived from three adjacent slopes in a Missouri oak–hickory forest and reciprocally planted in those sites. Microhabitats consisted of lower west‐facing slope (LW), south‐facing slope (SW), and north‐facing slope (NO). (Taken from Sork et al. 1993. American Naturalist).

Early in my career as a plant biologist, I was inspired by the classic work of Clausen et al. (1947) who showed that plants grew best when put near their native environments and that they grew better in those sites than plants derived from sites at other elevations. That work provided elegant proof of local adaptation, but, of course, the origins of that geographical differentiation could be explained by both natural selection on local populations and restricted gene flow from populations at different elevations. Several papers based on populations at the boundaries of mines with toxic metals showed that short‐lived plant populations could differentiate rapidly due to strong selection (Antonovics and Bradshaw 1970; Antonovics 1971), but it was less clear whether highly outcrossed tree populations could differentiate on such a local scale. After reading Slatkin's papers on gene flow (1973, 1985, 1987), I wondered whether selection could be strong enough to overcome gene flow from adjacent populations of highly outcrossing tree species. Forest geneticists have utilized provenance studies to illustrate regional differences across tree populations (Mátyás 1996). However, I questioned whether local adaptation could occur in tree populations on such a fine geographic scale as the mine boundaries or whether gene flow would swamp adaptive divergence. To test this, I set up a field experiment in Missouri where I planted acorns from adult northern red oaks growing in adjacent microhabitats using a quantitative genetic design (Sork et al. 1993). Among many differences found among the first‐year seedlings, it was particularly noteworthy that percent leaf damage, an indicator of resistance to herbivores, was always lowest for seedlings grown on the microhabitat of their maternal seed tree (Fig. 2). Moreover, this difference among subpopulations also showed up when seedlings were planted in a common garden setting (Stowe et al. 1994). Although we cannot rule out maternal effects or other factors, these strong phenotypic differences on a scale less than a few hundred meters indicate the local selection pressures in tree populations could be sufficiently intense to overcome gene flow, or gene movement is sufficiently restricted that adaptation can evolve on a local scale.

A second major driver of my research program twenty years ago was to understand the impact of landscape alteration on genetic diversity of forest tree species. This work was stimulated by a multi‐investigator study of the impact of forest ecosystem management on biodiversity, called the Missouri Ozark Forest Ecosystem Project (MOFEP), and administered through the Missouri Department of Conservation (Brookshire and Hauser 1993). Given evidence that landscape fragmentation and reductions in population size could jeopardize gene flow and connectivity (for example, see Hamrick et al. 1991; Ledig 1992; Templeton and Georgiadis 1996; Nason and Hamrick 1997), my role was to assess whether forest management practices would have an impact on genetic diversity of tree populations in 200+ ha plots. In contrast to the other MOFEP studies that looked at immediate impacts of forest‐cutting practices on insect, bird, and mammal biodiversity (Brookshire and Hauser 1993; Brookshire et al. 1997), because of the longevity of trees, our project would not be able to measure changes in diversity for at least 50 years. Instead, we chose to investigate the impacts on contemporary pollen flow because any changes in gene flow at the present time would shape future diversity. However, at that time, gene movement was studied largely through paternity and maternity analysis (e.g., Devlin et al. 1988; Adams et al. 1992; Streiff et al. 1999), which was not feasible for continuously distributed tree populations on a landscape scale (Sork et al. 1999; Smouse and Sork 2004). This applied conservation science investigation required a method of studying contemporary gene flow that could be conducted across a large area and that could test specific hypotheses about the impact of landscape change on future genetic diversity. Fortunately, during my sabbatical at the National Center for Ecological Analysis and Synthesis, I was able to organize an NCEAS‐funded working group of population genetics experts that would address this issue.

## Contemporary gene flow in trees at a landscape scale

**Figure 3:**
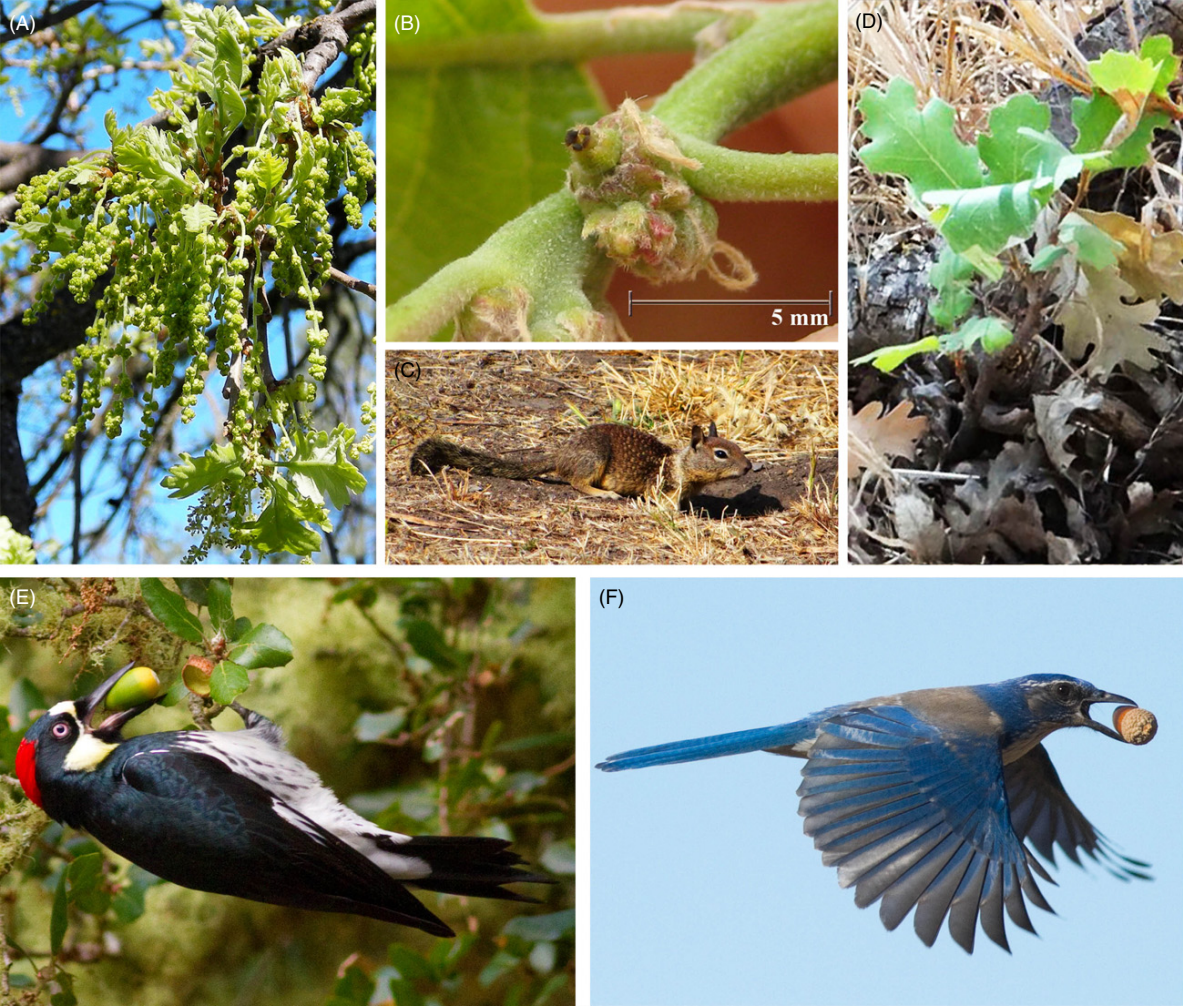
Stages of plant reproduction of wind‐pollinated and animal‐dispersed *Quercus lobata* involved in gene flow. (A) Emergence of male flowers (catkins) in February–April. (B) Subsequent emergence of separate female flowers with conspicuous stigma for pollen receipt. (C) The California ground squirrel, *Otospermophilus beecheyi*, is one of the several rodent seed predators that occasionally disperse acorns locally. (D) Postdispersal seedling establishment. (E) Acorn removal from tree by acorn woodpecker, *Melanerpes formicivorus*, followed by transport to granary for storage. (F) Long‐distance dispersal of acorn by western scrub jay, *Aphelocoma californica*, resulting in caching beneath soil. (Photos A–D by Andy Lentz ©; photos E–F by Marie Read ©).

Evolutionary biology has long inferred historical gene flow through genetic structure statistics (Slatkin 1985, 1987; Neigel 1997), but the study of contemporary gene flow allows an examination of the process of gene movement. Short distance gene movement creates neighborhoods (*sensu* Wright 1946), which sets up the opportunity for both genetic drift and natural selection to reduce the genetic variation. Long‐distance gene movement, on the other hand, maintains connectivity among tree populations. If gene flow is impeded by landscape changes, then a population becomes isolated and can decrease in effective size, which increases risk of inbreeding depression and loss of adaptive genetic variation through drift (Ledig 1992; Ellstrand and Elam 1993; Aguilar et al. 2008). The effects of landscape change may be mitigated in plant species with long dispersal kernels with fat tails, where gene flow will promote genetic diversity within local populations and connectivity among populations (Austerlitz et al. 2004; Smouse and Sork 2004; Sork and Smouse 2006). Indeed, the study of dispersal kernels has provided valuable detail about the processes of gene movement away from focal plants for many species (e.g., Austerlitz et al. 2004; Oddou‐Muratorio et al. 2005; Robledo‐Arnuncio and Gil 2005). However, my interest has been to test specific hypotheses about the genetic consequences of gene movement in different landscape contexts when locating all potential parents was not feasible. For this goal, new statistical approaches were developed through my collaboration with Peter Smouse (Rutgers University) and a series of talented younger scientists, including Frederic Austerlitz, Cyril Dutech, Rodney Dyer, Delphine Grivet, Jordan Karubian, Andrea Pluess, Juan Jose Robledo‐Arnuncio, and Douglas Scofield. Building on earlier work in Missouri Ozark Forests studying oak, pine, and flowering dogwood (Sork et al. 1997, 2005; Gram and Sork 1999, 2001; Dyer and Sork 2001; Apsit et al. 2002), we eventually focused our studies on the pollen and seed dispersal vectors of California populations of valley oak, *Quercus lobata* (Fig. 3). The overarching goals of this research are both to understand the processes of gene movement themselves from an evolutionary perspective and to provide a way to assess the extent to which they are influenced by landscape conditions.

### Pollen movement

For most tree populations, gene flow occurs primarily through pollen. When I first began this line of research, the primary method of studying pollen movement was to identify the paternity of progeny sampled from a maternal source (Meagher and Thompson 1987; Devlin et al. 1988; Adams et al. 1992; Streiff et al. 1999). While providing a very precise description of the pollen dispersal kernel, average dispersal distance, and the extent of long‐distance dispersal, its application is often limited to a single location, to a single year, and to a population where all of the adult parents could be identified completely or at least within a certain radius around a focal seed tree. For species with long‐distance dispersal or in difficult‐to‐sample habitats, paternity analysis is often not feasible (Sork et al. 1999; Smouse and Sork 2004; Sork and Smouse 2006). Moreover, the costs of replicating studies of paternity analysis where large numbers of progeny are needed relative to the number of pollen sources (Sork et al. 1999) make replication across sites and years difficult, feasibility issues aside. To find an alternative method, we changed our line of thinking: *What if we used the genetic structure of the pollen pool itself to make inferences about the effective number of pollen donors, the mean effective dispersal distance, and the effective neighborhood size (sensu Wright), and didn't worry about finding the pollen sources*? To do this, we developed a two‐generation procedure, dubbed twogener, which combines the simplicity of a population structure approach with the parent‐/offspring‐deductive aspects of the parentage approach (Smouse et al. 2001). Our approach emphasizes the genetic consequences of pollen movements rather than the description of each movement (Smouse and Sork 2004). A key parameter is the effective number of pollen donors (*N*_ep_). This number is typically much smaller than the total number of pollen donors because a few local pollen sources dominate, with rare long‐distance pollen flow from multiple different sources. From an evolutionary perspective, this result explains how local adaptation is compatible with long‐distance gene flow. From a conservation standpoint, estimating *N*_ep_ focuses on the diversity of the pollen pool as a critical element for conservation strategies because it reflects risk of genetic drift.

In our first application of the methods, we found that the mean *effective* pollen dispersal distance of the white oak, *Quercus alba,* in the heavily vegetated Ozark forest was restricted to about 17 m and the effective number of pollen donors was equivalent to 8 individuals (Smouse et al. 2001). Applying the same analysis to *Quercus lobata* in a savannah setting, we estimated that the mean effective pollen dispersal distance was almost four times greater than the distance for *Q. alba* (~65 m), and only about two effective pollen donors (Sork et al. 2002). We recognize that these initial estimates underestimated the effective number of pollen sources and dispersal distance, but the breakthrough was the introduction of a parameter that would allow comparison of the relative impacts of forest versus savanna settings. Initially, these paradoxically small estimates surprised some molecular ecologists because paternity studies demonstrate long‐distance gene flow and many pollen sources. However, none of these findings are inconsistent with long‐distance dispersal or multiple matings; they simply reflect the high frequency of the short distance movements in leptokurtic kernels and the fact that our measure is an ‘effective number’ rather than a total number of all pollen sources, as we later illustrate (Pluess et al. 2009).

An advantage of the twogener framework was that it introduced a tool for testing hypotheses about gene movement that was previously lacking for conservation genetic studies. For example, in an investigation of the impact of landscape change on pollen dispersal in flowering dogwood, *Cornus florida,* we presented tests that demonstrated that the pollen pool structure of forest sites differed among three forest‐cutting regimes and that forest thinning promoted pollen movement (Sork et al. 2005). Following these initial studies, the TwoGener framework has evolved over time. We began by improving how we model the dispersal kernel (Austerlitz et al. 2004), and then we developed a new way to estimate effective pollen dispersal distance and effective number of pollen donors (Robledo‐Arnuncio et al. 2006, 2007). We have emphasized effective number of donors because this parameter provides a useful assessment of the opportunity for evolutionary change, the risks of genetic drift, and other measures of interest to evolutionary and conservation biologists. This parameter, which is related to the effective population size of population genetics and akin to the species diversity index of ecology, can be used to test for statistical differences across years, landscapes, or species.

### Seed movement at a landscape scale

Because the study of seed dispersal is hindered by the difficulties in tracking seeds when the parents cannot be located, we wanted to extend our pollen pool structure approach to seed dispersal. In parallel with our treatment of male parentage in pollen dispersal studies (Smouse and Robledo‐Arnuncio 2005), we have articulated the consequences of seed movement for the effective number of maternal parents (*N*_EM_) within local patches and for the local site (Grivet et al. 2005). Taking advantage of the fact that maternal tissue in seeds allows us to unambiguously identify the maternal genotypes (Godoy and Jordano 2001), we illustrated our efforts using the study system of acorn woodpeckers at the UC Santa Barbara Sedgwick Reserve (see Fig. 3E). Although woodpeckers are more likely to acts as seed consumers than dispersers, they transport acorns to ‘granaries’, which are storage sites often in the barks of trees, and can be considered to be seed pools (Grivet et al. 2005). Woodpeckers can fly kilometers on any given day, but our analyses showed that the effective number of maternal trees found in any single granary (each containing the combined collection from a single woodpecker family's efforts) is 2–3 local trees (Scofield et al. 2010; Scofield et al. 2011), as would be predicted by optimal foraging theory (Thompson et al. 2014).

In the acorn woodpecker study, maternal oak genotypes had been geolocated, so we knew not only who they were, but also where they were. However, even where the maternal locations remain opaque, maternal identification is still useful. A prime example can be illustrated by our study of seed dispersal by long‐wattled umbrellabirds in an Ecuadorian tropical forest (Karubian et al. 2010). Umbrellabirds are characterized by a lek mating system and feed preferentially on palm fruits during the lekking season. The male birds forage in groups and fly at increasingly long distances to find fruits before returning to their lek where the seeds pass through their system. Even without locating the seed source, by genotyping seeds collected in seed traps in leks, this project was able to demonstrate that the birds visited many trees in the region and went long distances to do so.

Eventually, we translated our effective number of parent parameters into α− and γ− diversity statistics so that evolutionary biologists and ecologists could talk the same language. In the first phase of development, we described genetic diversity within a pool or patch (α) and genetic diversity across patches within the area (γ). We presented an elaborated treatment of diversity statistics in an *American Naturalist* article (Scofield et al. 2012) where we added measures of divergence (δ) in seed sources among patches as an alternative to the β‐parameter used in ecological diversity studies and introduced more statistical tests that permit comparison of different dispersal agents. This study illustrated the new measures by comparing seed transport by acorn woodpeckers with umbrellabirds, two species with very different social organizations and foraging patterns. The analyses showed that the territorial woodpeckers visited significantly fewer maternal source trees than did lekking males and that the seed pools of different woodpecker families were significantly more divergent from each other than were those created by males in different adjacent leks. These findings suggest that umbrellabirds foraged in many palm trees, and that different males visited the same trees before bringing palm fruits back to their respective lekking sites.

Whether we refer to the number of effective seed sources per patch as *N*_EM_ or α‐diversity, it is valuable to be able to complement this information with direct documentation of seed movement. Taking advantage of the fact that first‐year valley oak seedlings remain attached to the maternally inherited acorn tissue and that we have a well‐mapped study site at the UC Santa Barbara Sedgwick Reserve, with every adult genotype identified, we were able to track seed dispersal into valley oak seedling patches away from an adult canopy. We observed that most of the seedlings came from nearby trees, but many were dispersed from a seed tree 1–4 kilometers away, putatively by scrub jays (see Fig. 3F). Although the local diversity of those seedling patches was small, those rare long‐distance events demonstrate that seed dispersal can also contribute to long‐distance gene flow in oaks. In fact, in a separate study of the Mexican oak *Quercus castanea*, we found evidence of high seed‐mediated connectivity in a fragmented landscape (Herrera‐Arroyo et al. 2013). Such studies have illustrated both our novel methods and specific insight about gene flow in bird‐dispersed tree species.

### Joint impacts of pollen and seed movement

Because gene flow in plant populations is sequential, involving pollen dispersal followed by seed dispersal, each can have a separate contribution to the genetic diversity of the seedling population. However, seed dispersal has a disproportionate impact on the local effective size of recruiting populations because it moves both maternal and paternal alleles, whereas pollen only moves the male alleles (Crawford 1984; Hamilton and Miller 2002; Garcia and Grivet 2011). Estimating the separate contributions of pollen and seeds to gene flow has been elusive because many studies compare the two processes using different sets of individuals or markers. To overcome that limitation, we compared haploid pollen and diploid seed dispersal using maternally inherited tissue in the acorn seed coats attached to the seedlings and biparentally inherited leaf tissue from the same seedlings (Grivet et al. 2009), which can be performed even for incomplete genotypes resulting from DNA degradation in buried acorns (Smouse et al. 2012). To assess the respective contributions of pollen and seed flow, we introduced a novel indirect assessment of the separate male and female gametic contributions to total effective parental size (*N*_e_), based on kinship coefficients (Grivet et al. 2009; Robledo‐Arnuncio et al. 2012). Here, we demonstrated that the effective number of parents of dispersed seedlings (*N*_e_ = 6.7) was greater than nondispersed seedlings (*N*_e_ = 3.6), largely because the nondispersed seedlings had only one maternal source. Thus, we provided important new evidence that seed dispersal has a substantial impact on the local neighborhood size of newly established seedlings despite the homogenizing contribution of the pollen donors (Grivet et al. 2009).

The next development in our contemporary gene flow studies was to assess the genetic diversity of the male and female haploid gametic contributions to the seedling pools (Sork et al. 2015). This study utilized the diversity indices introduced in Scofield et al. (2012) to compare allelic diversity resulting from pollen and seed movement rather than estimates of the effective number of pollen or seed sources. Allelic diversity is estimated at the patch level (α−diversity) and site level (γ‐diversity). β‐diversity (=γ/α) represents the turnover in diversity across patches and is an estimate of the effective number of patches for the site. When the allelic composition of patches is similar across the site, β‐diversity is low, while divergent allelic composition among patches leads to high β‐diversity. As before, we found that α−diversity resulting from seed dispersal was much less than that from pollen dispersal within patches. A key difference between this studies with the Grivet et al. (2009) study of effective parental size is not the switch to diversity parameters, but the direct comparison of the haploid genetic contribution of pollen sources with the haploid contribution of the seed sources. Here, we show that female gametes still contribute less to the γ‐diversity of seedling populations than do male gametes, without the confounding inclusion of the diploid genotype.

Both studies demonstrate similar patterns of contemporary gene flow in oaks, but the diversity indices allow more dimensional analysis of the consequences of gene movement. First, despite long‐distance seed dispersal in valley oak, female gametes do not contribute as much to seedling genetic diversity as do wind‐dispersed male gametes. The difference is likely due to the fact that jays move acorns from a limited number of trees, even when they move them long distances. Second, the dispersal of male gametes maintains both the local diversity and connectedness of the regional gene pool. Third, the examination of γ‐diversity adds an important element to the assessment of the genetic consequences of dispersal, because it provides an effective way of summarizing accumulated genetic diversity across seedling pools within an area.

An additional contribution of these papers is the introduction of statistical tests to assess differences in diversity indices between sites or conditions. In general, this approach provides a framework to test the impact of changes in ecological circumstances created by a loss of a pollinator or dispersal agent or landscape alteration. There are three possible cases and the use of statistical tests will determine whether the α‐/γ‐diversity parameters differ significantly within each: (i) γ‐diversity does not change, but the α− versus γ‐diversity does; (ii) the α‐ and β‐diversity values are not affected, but the overall γ‐diversity is changed; (iii) α‐, β‐, and γ‐diversity measures are all affected. If statistical tests show that a disturbance affects cases (ii) or (iii), one can reliably conclude that the disruption is jeopardizing genetic diversity.

## Geographic patterns of genetic variation

The geographical patterns of genetic variation in a species are the outcome of demographic history of population expansion and contraction, migration history, and the impact of natural selection both spatially and temporally. By putting genes on a map, it is possible to extract inference on these processes from the geographical patterns of allele frequencies. The analysis of spatial patterns is a form of discovery‐based science that provides insight into the environmental context of the organism and its evolution. With my early ecological training, understanding the environmental context of my study systems is inherent in how I think about evolution. My research has always utilized a spatially explicit framework, and the landscape context can play such an important role in the distribution of genetic variation. This work has been done in collaboration with a geographer and plant ecologist, Frank Davis, a forest geneticist, Robert Westfall, postdocs, Cyril Dutech, and especially Delphine Grivet and Paul Gugger. Our studies fall into three types of approaches: spatial autocorrelation, geographic and climate associations of genetic gradients, and phylogeography.

### Spatial autocorrelation

The simplest form of spatial analysis of genetic variation is to examine the scale of spatial autocorrelation of alleles, particularly appropriate for examination of the extent of gene dispersal. Our early work in this arena involved a study of seed dispersal by tropical avian frugivores (Loiselle et al. 1995), in which John Nason developed a clever statistical parameter that transforms the autocorrelation value across distance classes into a kinship estimate. In a separate study of fine‐scale genetic structure of adult valley oak (Dutech et al. 2005), we found significant spatial structure in adults that was on a scale greater than contemporary gene flow, which provided evidence that the local populations have sufficient spatial structure to allow the evolution of local adaptation. In a more recent study (Sork et al. 2015), we were able to dissect the male and female gametic contributions to the fine‐scale genetic structure of oak seedlings to demonstrate that the male gametes reduce structure while the female gametes produce it. Because it is not always possible to directly observe gene dispersal, spatial autocorrelation analyses provide valuable insight about the genetic consequences of propagule movement. Most plant populations show some degree of spatial genetic structure (SGS), suggesting this short distance gene flow creates opportunity for the survival of locally adapted genetic variation or that local adaptation might even promote SGS.

### Geographic and environmental associations with genetic gradients

A key limitation of spatial autocorrelation analysis is that it does not incorporate spatially explicit environmental factors associated with geographic location. When a species is widely distributed, its evolutionary history can result in a geographic mosaic of genotypes that reflects the movement of genes, the impact of genetic bottlenecks, and the effects of local selection pressures. We can use that mosaic to infer evolutionary history and the processes that have shaped the geographic patterns (Avise 2000) or for conservation purposes (Moritz 1995). For example, Richard Rayburn, a former resource manager for California State Parks, and Craig Moritz proposed a conservation strategy of prioritizing the protection of regions that are ‘evolutionary hotspots’ (Rayburn and Moritz 2006). To identify the regions of conservation importance for valley oak, we used a multivariate approach that we had previously used to analyze the environmental correlations with geographic patterns of genetic variation in Missouri Ozark forests (Gram and Sork 1999, 2001). Multivariate models, such as canonical correspondence analysis (CCA), are useful because they detect small genetic differences across loci that reveal cumulative patterns of differentiation that are not apparent with analyses of one locus at a time (Conkle and Westfall 1984; Kremer and Zanetto 1997) and provide signatures of genetic differences that can be correlated with environmental gradients or associated with geographic regions. In one study, we used the multilocus genotypes of valley oak as indicators of unique genetic composition and demonstrated how one could design a reserve network to maximize the preservation of unique genetic diversity for the species (Sork et al. 2009). In another study, we analyzed the geographic patterns of multilocus chloroplast and nuclear microsatellite genetic markers of individuals distributed widely across the valley oak range. This allowed us to identify the areas with distinctive histories and genetic composition that should be given priority in reserve network design (Grivet et al. 2008). Sadly, two of the regions we identified lay in areas of high human development and little protection where habitat loss has already been severe. Thus, this conservation genetic analysis revealed that without a careful preservation plan, valuable evolutionary information will be lost for valley oak and that efforts to preserve populations in this area will be particularly important.

Given concerns about the impact of rapid climate change on long‐lived species, the association of genetic variation with climate can provide a map of regional differences in genetic variation that could indicate how regions might respond differently to changes in climate. This topic is particularly relevant for trees that were established under climate conditions several hundred years ago. In a species‐wide analysis of genetic variation in valley oak, we found the geographic structure of multivariate genotypes is highly correlated with climate variables (Sork et al. 2010), which was initially surprising given that putatively neutral markers were used. However, the patterns make sense if climate shapes evolutionary processes, such as migration or population expansion and contraction, which in turn affect genome‐wide patterns of variation. This study employed climate niche modeling (Maximum Entropy, MAXENT) based on downscaled historical (1971–2000) and future (2070–2100) climate grids to illustrate how geographic genetic variation could interact with regional patterns of 21st century climate change. Our models predicted that future climate niches for valley oak will shift in location due to changes in climate. In some regions, the climate niches of local populations might show local displacement of only a few meters to hundreds of meters, while in others, they might shift 60–100 kilometers. Thus, for several populations, it is unlikely that the movement of genes through pollen dispersal or colonization events through seed dispersal will be great enough to allow the spread of valley oak to more suitable sites. This work predicts that the late 21st century climate conditions could lead to regional extinctions for many valley oak populations unless they have preexisting tolerance for warmer, drier, and more seasonal climate environments. In fact, for some tree species, interventions, such as assisted migration, may be necessary (Aitken and Whitlock 2013).

### Phylogeography

Phylogeography is the study of geographic patterns of genetic lineages with the goal of understanding how the physical landscape and climate cycles shape the evolutionary history of species, the emergence of new species, and the extinction of others (Avise 2000). Through my team's phylogeographic studies of valley oak and other species in California (Grivet et al. 2006; Sork et al. 2010; Gugger et al. 2013; Ortego et al. 2014; Sork and Werth 2014; Werth and Sork 2014), we have shown that the lack of recent glaciation in much of California and the overwhelming majority of the valley oak species range has allowed the accumulation of high genetic diversity and long‐term persistence of local populations. For example, we learned that valley oak has much greater chloroplast diversity than European oaks, whose range has experienced recent glaciation (Grivet et al. 2006). In Gugger et al. (2013), we found genetic divergence between eastern and western subpopulations of valley oak that took place approximately 150 000 years ago during the last interglacial warming. This estimate means that contemporary oak populations are at least several hundred thousand years old, which would explain why they have more chloroplast haplotype diversity than European oaks. We have also shown that these haplotypes are highly localized (Grivet et al. 2006; Gugger et al. 2013), indicating that colonization via seeds has remained local over extended periods. In fact, Gugger et al. (2013) found that the current distribution of genetic variation retains a signature of climate association with the time of the Last Glacial Maximum, less than 20 000 years ago. Whether that residual pattern represents adaptation remains an open question. Overall, these findings provide valuable background on the evolutionary history of valley oak. In addition, our work on valley oak and other California species contributes to the growing body of literature documenting California as a hotspot of biodiversity (Raven and Axelrod 1978; Calsbeek et al. 2003; Rissler et al. 2006; Lancaster and Kay 2013).

## Landscape genomics of adaptive genetic variation

For populations of valley oak, a tree found in the rainy northwestern part of its range is likely to have a different genetic composition than one from the warm, dry south. This pattern could be true due to isolation by distance factors alone. However, I am especially interested in the question: To what extent are trees locally adapted and, in particular, at what spatial scale is valley oak adapted to its local climate environment? Given that the generation time of a valley oak is on the order of 100 years, it seems unlikely that local populations will evolved to keep pace with the current rapid rate of climate change. However, some populations may have preexisting tolerance to hotter, drier climates with more seasonality, while other populations may not. Thus, identifying geographic patterns of adaptive genetic variation to prioritize regions for conservation is a topic of concern to many forest geneticists (Neale 2007; Aitken et al. 2008; Alberto et al. 2013; Sork et al. 2013; Kremer et al. 2014).

With the emergence of next‐generation sequencing for nonmodel systems, our research group is characterizing geographic patterns of adaptive genetic variation associated with response to climate. Recently, I have participated in working groups that have emphasized genomic research as a high priority for understanding the evolutionary processes in natural populations (Andrew et al. 2013) and for developing new genomic tools for managing forest tree populations (Sork et al. 2013), especially oaks (Petit et al. 2013). This new phase of my research over the last five years has benefitted from discussions with forest geneticists Sally Aitken, David Neal, and from collaborations with Andrew Eckert, Matteo Pellegrini, Alex Platt, Jessica Wright and in particular, my postdoc Paul Gugger. Our joint long‐term goal is to understand the basic evolutionary biology of local adaption in tree populations, particularly in response to climate, and the extent to which the genetic composition of tree populations will shape their response to climate. I have multiple projects underway using several genomic approaches, but here, I will present two initial studies. In the first, we utilized DNA sequence data from specific genes with functions related to climate response and applied a landscape genomic approach to associate genetic gradients with climate gradients as evidence for selection (Sork et al. 2016). The second study, we compared genetic and epigenetic differences across populations to see whether methylated DNA sequences could be important in shaping phenotypic variation in response to climate (Platt et al. 2015).

### Climate‐associated candidate genes

**Figure 4:**
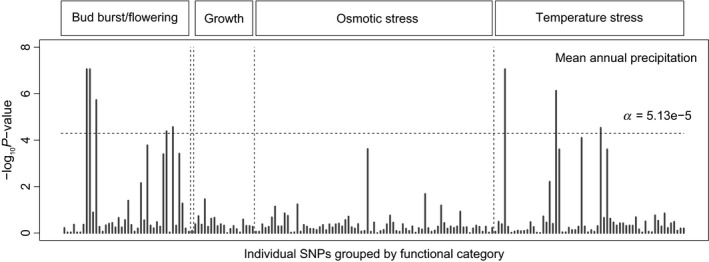
Manhattan plot showing environmental association analysis of 195 *Q. lobata* SNPs found within four functional gene categories versus mean annual precipitation. Outlier SNPs with significant correlations with climate variables are detected by plotting the negative log probability of SNP–climate association. We found significant correlations with mean annual precipitation only. The dashed line indicates the significance threshold after correcting for multiple tests. (Modified from Sork et al. 2016. American Journal of Botany).

In designing my first study of adaptive genetic variation, I was concerned that gene flow in oaks was so extensive that the signal of genes under selection would be weak. So, we identified 40 genes within four functional categories for response to climate that could be potential candidates under selection: bud burst and flower timing genes, growth genes, osmotic stress genes, and temperature stress genes (Sork et al. 2016). Then, we sequenced individuals from thirteen localities throughout the species range. Based on 195 single‐nucleotide polymorphisms (SNPs) across the 40 genes, we identified outlier SNPs likely to be under selection through several approaches. First, we found that the top 5% of *F*_ST_ estimates ranged from 0.25 to 0.68, while the mean *F*_ST_ = 0.03. Second, in correlation analyses of the 195 SNP frequencies with climate gradients, we found three SNPs within bud burst and flowering genes and two SNPs within temperature stress genes that were significantly associated with mean annual precipitation (Fig. 4). Finally, given that adaptive variation is often polygenic and climate niche is multivariate, we used a canonical correlation multivariate model to examine this highly complex story for each of the four functional sets of genes. Many of the SNPs identified through the single‐locus models above were again outliers in these multivariate models, but we also found additional SNPs of smaller effects in growth genes that may be under selection. These analyses revealed that 10 of the 40 candidate genes showed evidence of spatially divergent selection (Sork et al. 2016), an exciting finding because it demonstrates the potential of landscape genomic studies to detect adaptive genetic variation in a nonmodel systems.

Future landscape genomic work should include a random and much larger sample of SNPs that will allow for a good estimate of background genetic structure that controls for the confounding effects of demographic history (Coop et al. 2010; Lotterhos and Whitlock 2014, 2015). Ideally, landscape genomic studies should include as many individuals and localities as possible to increase the power to detect climate associations. The use of functional genes increases the efficiency of finding outliers, but it also presents an ascertainment bias by not testing all potential genes. Alternatively, one can use a genome‐wide method to randomly subsample the genome, such as RADseq (Baird et al. 2008) or GBS (Elshire et al. 2011) that could find both neutral and adaptive variants, but this methods utilizes only a small percent of the genome (often <5%) and could miss more genes under selection. Some studies include both candidate genes to see whether they show spatially divergent selection and randomly selected SNPs to describe demographic history (Keller et al. 2012; De Kort et al. 2014). As the costs of sequencing decline, whole‐genome sequencing will be an option for genome‐wide association studies (GWAS) designed to identify the important environmentally associated candidate genes that might be responsible for tree performance and survival.

### Epigenetic differentiation in valley oak

Epigenetic variation has the potential to shape phenotypic plasticity and adaptation to the environment, each of which could be particularly important for the persistence of long‐lived organisms such as trees (Franks and Hoffmann 2012; Braeutigam et al. 2013). This potential is illustrated by studies of natural populations of *Arabidopsis thaliana* that reveal patterns of DNA methylation which vary across environments and may provide a mechanism to enhance the adaptation to the local environment (Schmitz 2014). Evidence from tree populations illustrates one epigenetic mechanism, DNA methylation of cytosine residues, which has all the ingredients to be involved in evolution by natural selection in plants. It is sometimes heritable across many generations (Becker et al. 2011; Schmitz et al. 2011; Zhang et al. 2013), among individuals, and among populations (Herrera and Bazaga 2010, 2011; Schmitz et al. 2013). Mounting evidence suggests DNA methylation may also underlie trait variation by affecting gene expression when found in regulatory or genic regions. For example, flowering time in *Arabidopsis* (Soppe et al. 2000), floral symmetry in *Linaria* (Cubas et al. 1999), and fruit ripening in *Solanum* (Manning et al. 2006) are partially under epigenetic control by cytosine methylation.

**Figure 5:**
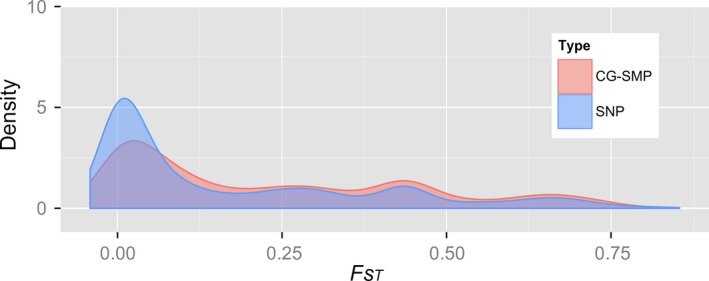
Densities of *F*_ST_ values for CG‐single methylated polymorphisms (CG‐SMP) and single‐nucleotide polymorphism (SNP) across three populations of *Quercus lobata* are shown for the range of *F*_ST_ values. (Modified from Platt et al. 2015. Molecular Ecology).

To test whether epigenetic variation differs across populations of valley oak, we sampled individuals from three southern populations with divergent elevations, levels of rainfall, and seasonality of rainfall (Platt et al. 2015). We then sequenced bisulfite‐treated reduced‐representation ‘genomes’, generating 100 base pair sequence fragments from randomly selected loci across the genome and converting unmethylated cytosines to thymines. Cytosine methylation occurs in three DNA sequence contexts, CG, CHG, and CHH (where H is non‐G), each of which has different regulatory pathways (Law and Jacobsen 2010) and thus potentially differing roles in adaptation. We found that CG sites, which are commonly found in genic regions (Cokus et al. 2008; Takuno and Gaut 2013), were the most highly methylated and, on average, showed much more differentiation across populations than either CHG or CHH sequences. To assess whether the SNP (single‐nucleotide polymorphism) and SMP (single methylated site polymorphism) variation differs across populations, we calculated population differentiation, *F*_ST_, at each of the polymorphic loci as a measure of the extent to which each polymorphism is distributed nonrandomly across populations. Typically, tree population genetic studies using putatively neutral, nuclear microsatellite markers report *F*_ST_ values in the range of 0.08–0.20 (Loveless and Hamrick 1984). Similarly, in a study of range‐wide populations of valley oak, we found *F*_ST_ **= 0.12 for microsatellite loci (Grivet et al. 2008). In contrast, Platt et al. (2015) found that mean epigenetic differentiation among three populations at variably methylated CG sites (CG‐SMPs) was higher than that based on SNPs (*F*_ST_ = 0.28 versus *F*_ST_ = 0.19, respectively) and that the distribution of CG‐SMP *F*_ST_ values included more values at the upper end of the distribution (Fig. 5). The findings of this study provide evidence that divergent selection and local adaptation are operating on portions of the valley oak genome marked by CG‐SMPs. While we do not yet know the mechanisms creating such high levels of epigenetic differentiation among populations, recent work on Norway spruce shows that temperature during zygote formation appears to create an epigenetic ‘memory’ in the progeny that regulates bud phenology and cold acclimation later on in life (Yakovlev et al. 2011). It will be necessary to combine studies that associate epigenetic variation with environmental and phenotypic variation with studies that look at the epigenetic effects on those phenotypes, if we are to assess the extent to which epigenetic processes play a role in either phenotypic plasticity or local adaptation. The role of DNA methylation in shaping phenotypes that enhance tolerance to local environments is a long way from being understood, but this study is a good start to assessing the extent of epigenetic differentiation in natural tree populations.

## Summary and conclusions

1. The interaction of pollen and seed dispersal vectors with landscape features shapes contemporary gene flow. My research has shown that pollen movement in oaks is responsible for maintaining connectivity among populations, yet local pollination is sufficiently frequent that it is possible to create fine‐scale genetic structure that could facilitate local adaptation. Seed dispersal is more likely to be restricted, so it accounts for much more of the fine‐scale genetic structure than does local pollen movement. Dispersal agents, such as western scrub jays, facilitate long‐distance acorn movement that would allow colonization of new sites, but because they visit few trees, the genetic diversity of patchy seedling populations is reduced during this phase from that introduced through pollen dispersal. The generality that has emerged from this work and that of others for many tree species is that localized gene movement of pollen and seeds will create sufficient genetic structure to allow the evolution of local adaptation, while long‐distance gene flow will maintain high levels of genetic diversity and spread favorable alleles over large spatial scales.
2. The analysis of contemporary gene flow in plants provides valuable information on the extent to which human disturbances are jeopardizing future genetic diversity by isolating populations. However, conservation scientists need to test hypotheses about the impact of human disturbance. My collaborators and I have also introduced statistical tests for landscape scale questions to determine whether landscape change through fragmentation isolates or enhances gene movement or whether dispersal vectors differ in their impact on the genetic structure of populations and thus their loss would jeopardize genetic diversity. Thus, our studies are part of a growing body of literature in landscape genetics exploring whether plant populations are threatened by human activities.
3. The geographic patterns of genetic variation reflect the evolutionary history of a species that is the foundation for current responses to environmental conditions. Valley oak populations are likely to be several hundred thousand years old with high genetic diversity within populations and high gene flow among populations. While nuclear markers indicate extensive connectivity across the species range, chloroplast haplotypes indicate that colonization is local. This restricted dispersal would allow evolution of local adaptation. In addition, the species‐wide spatial pattern of genetic variation reflects an influence of climate through either gene flow or selection against migrants into populations. Regardless, this pattern explains the prevalence of genetic and phenotypic differences among tree populations shown here and which is likely to be true for many forest tree species.
4. The emergence of next‐generation sequencing tools now allows the direct analysis of candidate genes and epigenetic impacts that could shape phenotypic response to the environment. Our work demonstrates extensive genetic differentiation across populations for certain genes and a correlation between genetic and climate gradients that provides initial evidence of local adaptation. However, future work needs to also control for background genetic structure that may be confounded with environmental associations. Our evidence of significant epigenetic differentiation at methylated sites further provokes the question of the extent to which epigenetic processes might shape phenotypic response to local environments.
5. We have also demonstrated that the homogenizing influences of long‐distance gene flow are not sufficient to ‘swamp out’ the impact of selection on tree populations. Instead, genes show a strong impact of selection likely due to both the strength of selection and the presence of fine‐scale genetic structure.
6. Valley oak populations have already experienced a reduction in regional coverage due to habitat transformation and problems with regeneration. Restoration efforts will need to consider future climates as they consider the impact of climate change on the future environments of their restoration sites. It is my goal to develop and ‘ground‐truth’ landscape genomic approaches that can be used to inform future conservation strategies when long‐term common garden and reciprocal transplant experiments are not feasible. This information will not only help to preserve this and other California oaks, but also provide a case study for other tree species of conservation concern.

## Future directions

My research efforts have taken place in the larger context of landscape genetics and genomics, plant evolutionary biology, forest genetics, and conservation science. My studies are built around the valley oak study system is poised to complement other exciting forest genetic research to address the emerging questions about the ability of long‐lived species to face and survive anthropogenic challenges. Maintaining healthy tree populations is not only important to their survival, but also important to sustaining the ecological processes of the ecosystems they define (Hughes et al. 2008; Schoener 2011).

1. *Gene movement on the landscape*. Unlike studies of animal landscape genetics that have focused on the impact of landscape features on migration, landscape genetic studies of plant populations initially focused more on geographical and environmental associations of genetic variation (Manel et al. 2003, 2010; Storfer et al. 2007), than on gene movement (with some notable exceptions, such as McRae 2006; Dyer et al. 2012). With the availability of genomic tools for nonmodel systems, the next frontier is the multidisciplinary analysis of gene movement to detect the adaptive genetic variation using large numbers of SNPs that permit the simultaneous analysis of historical migration and demographic events (Sork and Waits 2010; Manel and Holderegger 2013; Sork et al. 2013). Using species‐wide samples of valley oak, we currently have a landscape genomic study underway based on GBS to assess the extent to which neutral versus selective processes are shaping the geographic structure of genetic variation.
2. *Evolution of local adaptation*. To understand how local adaptation evolves, we need first to identify candidate genes underlying phenotypic traits under selection and the fitness consequences of those traits (Mitchell‐Olds et al. 2007; Stapley et al. 2010; Anderson et al. 2011; Strasburg et al. 2012). Landscape genomic analysis using *F*_ST_ outlier tests or environmental association analysis is one way to identify the candidate genes for local selection, when controlling for demographic history (for example, see Eckert et al. 2010; Holliday et al. 2010; Keller et al. 2012). For forest trees, the GWAS approach can be a useful way to detect candidate genes associated with phenotypes (Nichols et al. 2010). However, additional work in evolutionary and quantitative genetics may be needed to document the functions of those genes. Alternatively, experimental studies examining gene expression under different environmental conditions could be used to identify the number of genes and the gene networks responsible for phenotypes that might be under selection (Alberto et al. 2013; De Kort et al. 2014). Eventually, experiments will be necessary to test the adaptive consequences of individual alleles detected through these approaches (Barrett and Hoekstra 2011). In short, the breakthroughs in understanding local adaptation will come from an integrative approach. In our research program, we plan to integrate findings from a variety of approaches: environmental associational analysis of landscape genomic studies, studies of phenotypes measured a recently established long‐term provenance study for valley oak (Delfino‐Mix et al. 2015), studies of gene expression and physiological response in drought‐treated oak seedlings (Peñaloza Ramírez, Gugger, Wright, Sork, unpublished data; Steele, Sweet, Davis, Sork, unpublished data) that utilize our reference transcriptome (Cokus et al. 2015), and the production of a high quality gene sequence of valley oak to facilitate our ability to identify the climate‐associated genes and their genetic architecture (Sorel, Langley, Pellegrini, Sork, Salzberg, work in progress).
3. *Evolutionary and ecological response to climate change*. How plants are going to respond to rapid climate change is a critical issue (Parmesan 2006; Anderson et al. 2012). For long‐lived trees that became established under very different climate conditions and have a generation time that hampers rapid evolution, this concern is particularly salient (Savolainen et al. 2007; Aitken et al. 2008; Kremer et al. 2012; Alberto et al. 2013). Are most tree species resilient to a broad range of climate conditions (Hamrick 2004)? Is local adaptation sufficiently strong that we will need to assist migration across populations (Aitken and Whitlock 2013)? Will plant response require the evolution of new locally adapted phenotypes with existing or new genetic variation (Stapley et al. 2010)? Does epigenetic variation offer a mechanism for trees to respond ecologically to climate (Braeutigam et al. 2013)? My long‐term goal is to use valley oak as a case study of how a long‐lived tree species can respond to climate change. Besides the information generated for this particular species, we hope to assess the extent to which we can map specific genes and identify regional populations of concern to design management strategies for the maintenance of healthy forests.

In short, the integration of 21st century genomic approaches, quantitative and population genetics, and recently developed geographic methods of modeling environmental data provides exciting and unprecedented opportunities to understand the evolutionary and ecological processes of plants and important applications.

## Data archiving statement

No new data are reported in this manuscript. Archived data details are available through original publications.
